# Nepheliosyne B, a New Polyacetylenic Acid from the New Caledonian Marine Sponge *Niphates* sp

**DOI:** 10.3390/md11072282

**Published:** 2013-06-27

**Authors:** Nathalie Legrave, Souhir Hamrouni-Buonomo, Maeva Dufies, Vincent Guérineau, Jean Vacelet, Patrick Auberger, Philippe Amade, Mohamed Mehiri

**Affiliations:** 1 Chemistry Institute of Nice (ICN), UMR 7272 CNRS, Bioactive Molecules, University of Nice-Sophia Antipolis, ParcValrose, F-06108 Nice Cedex 02, France; E-Mails: nathalie.legrave@unice.fr (N.L.); souhirhamrouni@yahoo.fr (S.H.-B.); philippe.amade@unice.fr (P.A.); 2 UR Ecosystems and Aquatic Ressources (UR03AGRO1), Agronomic National Institute of Tunisia (INAT), Carthage University, 43 Avenue Charles Nicolle, Tunis 1082, Tunisia; 3 Mediterranean Centre of Molecular Medicine (C3M), INSERM UMR 1065, Team 2: Cell Death Differentiation and Cancer, Batiment ARCHIMED, 151 Route de Saint-Antoine de Ginestière, BP2 3194, 06204 Nice Cedex 3, France; E-Mails: maeva.dufies@gmail.com (M.D.); patrick.auberger@unice.fr (P.A.); 4 Gif Research Centre, Chemistry Institute of Natural Substances, CNRS Avenue de la Terrasse, 91198 Gif-Sur-Yvette Cedex, France; E-Mail: vincent.guerineau@cnrs.fr; 5 Aix-Marseille University, CNRS UMR 7263, Mediterranean Institute of Biodiversity and Marine and Continental Ecology (IMBE), Station Marine d’Endoume, Rue de la Batterie des Lions, Marseille 13007, France; E-Mail: jean.vacelet@imbe.fr

**Keywords:** marine natural products, highly oxygenated polyacetylenes, marine sponges, Porifera, Haplosclerida

## Abstract

A new C_47_ polyoxygenated acetylenic acid, nepheliosyne **B** (**2**), along with the previously described nepheliosyne **A** (**1**), have been isolated from the New Caledonian marine sponge *Niphates* sp. Their structures have been elucidated on the basis of extensive spectroscopic analyses. These metabolites exhibited a moderate cytotoxicity against K562, U266, SKM1, and Kasumi cancer cell lines.

## 1. Introduction

Several natural acetylenic metabolites, often featuring a polyketide or fatty acid origin, have been isolated from plants, fungi, marine algae, sponges, and tunicates [1]. The main source of oxygenated polyacetylenes is the phylum Porifera [2], especially the genera *Xestospongia* [3,4], *Petrosia* [5,6,7,8,9], and *Haliclona* [10,11,12]. The structure of these metabolites differs significantly from each other in terms of chain-length, functional groups as well as their locations in the carbon framework. Nepheliosyne **A** [3], petrosolic acid [6], osirisynes **A**–**F** [10], haliclonyne [11], and fulvynes **A**–**I** [12] are a few examples of linear acetylenes with a diacetylenic carbinol and an α-yne carboxylic acid functionality as common structural features. Several of these sponge-derived polyacetylenes exhibit potent bioactivities including antiviral [6], antimicrobial [7,12], cytotoxic [8,9], and enzyme inhibitory [13] activities. Nepheliosyne **A** and petrosolic acid, isolated from *Xestospongia* sp. and *Petrosia* sp., respectively, are the only examples of oxygenated polyacetylene with diacetylenic carbinol, α-hydroxyketone, and α-yne carboxylic groups. Some of the reported data of nepheliosyne **A** remain hypothetical, specifically the size of the methylene link between the two groups α-yne carboxylic and α-hydroxyketone. 

In the course of our search for bioactive marine natural products, we have investigated the New Caledonian marine sponge *Niphates* sp. (Haplosclerida, Niphatidae). In this paper, we report the isolation and structural elucidation of new polyhydroxylated acetylene, nepheliosyne **B** (**2**), along with nepheliosyne **A** (**1**), previously isolated from the marine sponge *Xestospongia* sp., whose structural elucidation is herein completed. We also report their cytotoxic properties against K562, U266, SKM1, and Kasumi cancer cell lines.

## 2. Results and Discussion

**Figure 1:**
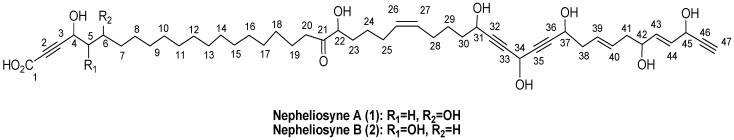
Structure of nepheliosynes **A** (**1**) and **B** (**2**).

The CH_2_Cl_2_/MeOH (1:1, v/v) crude extract of *Niphates* sp. was fractionated by Flash Vacuum Liquid Chromatography, eluting with a gradient of decreasing polarity from H_2_O to MeOH. The subsequent MeOH fraction was purified by semi-preparative reverse-phase HPLC (Phenomenex Luna C18, 250 × 10 mm id, 5 μm, gradient H_2_O/MeCN/Formic Acid 50:50:0.1 to 0:100:0.1) to afford pure nepheliosyne **A** (**1**) (8.7 mg) and nepheliosyne **B** (**2**) (6.7 mg) (Figure 1).

**Figure 2:**
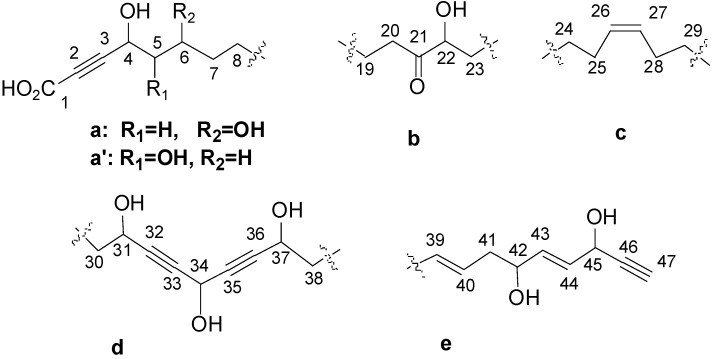
Partial structures (**a**, **a′**, **b**–**e**) of nepheliosynes **A** (**1**) and **B** (**2**).

Both compounds, obtained as amorphous solid, have the same molecular formula C_47_H_70_O_11_ deduced from the HR-MALDI-TOF data which showed for each a pseudomolecular ion adduct at *m*/*z* 833.4803 [M + Na]^+^. The IR spectrum showed bands at 3450, 3300, 2250, 2100, and 1705 cm^−1^ suggesting the presence of double bonds, triple bonds, and hydroxyl groups. A preliminary NMR spectral analysis showed similarities and strongly supported the presence of a polyhydroxylated acetylenic skeleton. The ^1^H and ^13^C NMR spectra of compound **1** (see Supporting Information and Table 1) revealed the following functional groups: (a) Seven non-protonated sp carbons (δ_C_ 87.3–78.0) and one terminal methine (δ_C_ 74.4; δ_H_ 2.90); (b) Six sp^2^ carbons (δ_C_ 135.7–127.5 and δ_H_ 5.91–5.38) suggesting three disubstituted carbon-carbon double bonds; (c) Eight oxymethine groups (δ_C_ 78.0 to 52.3, δ_H_ 5.17–3.79); and (d) A propargyl carboxylic group and a ketone identified from the carbon signals at δ_C_ 157.6 and 215.4, respectively. The relatively high field chemical shift of several of the eight oxymethine carbon resonances suggested their allylic and propargylic positions. The ^1^H and ^13^C NMR spectra of compound **2** (see Supporting Information and Table 2) allowed the identification of the same functional groups. These elements suggested partial structures **a** (for compound **1**), **a′** (for compound **2**), as well as the common parts **b**–**e** (Figure 2) to be identified as shown.

Fragment **a** in compound **1**, included two oxymethines at δ_H_ 4.64 (H-4, δ_C_ 59.3) and δ_H_ 3.84–3.79 (H-6, δ_C_ 68.4) linked together by a methylene at δ_H_ 1.86–1.71 (H_2_-5, δ_C_ 45.6). Proton at δ_H_ 3.84–3.79 (H-6, δ_C_ 68.4) was coupled to a methylene at δ_H_ 1.45 (H_2_-7, δ_C_ 38.7) in turn linked to another methylene at δ_H_ 1.30 (H_2_-8, δ_C_ 30.8–30.4). Heteronuclear Multiple Bond Correlation (HMBC) correlations were observed between, both H-4 (δ_H_ 4.64) and H-5 (δ_H_ 1.86–1.71) and C-3 (δ_C_ 87.3), and between H-4 and C-2 (δ_C_ 78.0) thus assigning the carbon resonances of the triple bond in fragment **a** (Table 1).

Fragment **a′** in compound **2**, was formed by two vicinal oxymethines at δ_H_ 4.26 (H-4, δ_C_ 67.1) and δ_H_ 3.58 (H-5, δ_C_ 74.9), itself coupled to methylenes at δ_H_ 1.69–1.44 (H_2_-6, δ_C_ 33.5) and at δ_H_ 1.29 (H_2_-7 and H_2_-8, δ_C_ 30.8–30.4). In a similar way than fragment **a** in compound **1**, HMBC correlations were observed between, both H-4 (δ_H_ 4.26) and H-5 (δ_H_ 3.58) and C-3 (δ_C_ 85.3), and between H-4 and C-2 (δ_C_ 80.1) thus assigning the carbon resonances of the triple bond in fragment **a′** (Table 2). The down-shifted proton value of H-4 in fragments **a** and **a′** suggested its connection to the α-yne carboxylic acid moiety that had to be the first terminal part of the chain.

**Table 1:** ^1^H NMR (500 MHz, CD_3_OD) and ^13^C NMR (125 MHz, CD_3_OD) data of nepheliosyne **A** (**1**).

Position	δ_C_ (ppm)/Multiplicity	δ_H_ (ppm)/ *J* (Hz)/Multiplicity	^1^H-^1^H COSY/TOCSY	^1^H-^13^C HMBC
1	157.7, qC			
2	78.0, qC			
3	87.3, qC			
4	59.3, CH	4.64, dd, 9.3, 3.5	5a, 6, 7	2, 3, 5, 6
5a	45.6, CH_2_	1.74–1.71, m	4, 5b, 6, 7	3, 4, 7
5b	45.6, CH_2_	1.86–1.81, m	4, 5a, 6, 7, 8	3, 4, 6, 7
6	68.4, CH	3.84–3.79, m	4, 5a, 5b, 7, 8	4, 5b
7	38.7, CH_2_	1.45, m	4, 6, 5a, 5b, 7, 8	8
8–18	30.8–30.4, CH_2_	1.30, m	5a, 5b, 7, 17, 19	
19	24.1, CH_2_	1.59–1.55, m	18, 20, 24, 25	20, 24, 25
20	38.6, CH_2_	2.61–2.49, m	18, 19, 25, 26	18, 19, 21
21	215.4, qC			
22	78.0, CH	4.05, dd, 8.1, 4.2	19, 23, 24	21, 23, 24
23a	34.6, CH_2_	1.74–1.69, m	22, 24, 25, 26	20
23b	34.6, CH_2_	1.61–1.49, m	22, 24, 25, 26	20
24	26.2, CH_2_	1.40–1.35, m	20, 22, 23, 25, 26	19, 23, 25, 26, 27
25	28.0, CH_2_	2.14–2.05, m	20, 23, 24, 26, 27	18, 19, 24, 26, 27
26	130.7, CH	5.38, m	20, 23, 24, 25, 28	25
27	130.9, CH	5.38, m	28, 29, 30, 31	28
28	27.8, CH_2_	2.14–2.05, m	26, 27, 29, 30, 31	26, 27, 29, 30
29	26.7, CH_2_	1.56–1.49, m	26, 27, 28, 30, 31	28, 30, 31
30	38.3, CH_2_	1.70–1.64, m	26, 27, 28, 29, 31	28, 29, 31
31	62.6, CH	4.33, dd, 6.7, 1.4	26, 27, 28, 29, 30, 34	30, 32, 33
32	83.1, qC			
33	82.9, qC			
34	52.3, CH	5.17, t, 1.6	29, 30, 31, 37, 38	32, 33, 35, 36
35	85.6, qC			
36	85.9, qC			
37	62.6, CH	4.36, dd, 6.6, 1.6	34, 38, 39, 40	35, 38
38	36.8, CH_2_	2.45, t, 6.1	31, 37, 39, 40	36, 37, 39, 40
39	129.2, CH	5.64–5.54, m	37, 38, 40, 41, 42	38, 42, 44
40	127.5, CH	5.64–5.54, m	37, 38, 39, 41, 42	41, 42, 44
41	36.3, CH_2_	2.38–2.25, m	39, 40, 42	39, 40, 42, 43
42	72.2, CH	4.15, dd, 6.3	39, 40, 41, 44	39, 40, 41, 43, 44
43	135.7, CH	5.91, ddd, 15.4, 5.9, 1.3	41, 42, 44, 45	42, 44, 45
44	130.7, CH	5.78, ddd, 15.4, 5.7, 1.2	41, 42, 43, 45	42, 43, 45, 46
45	62.5, CH	4.83, d, 5.5	37, 42, 43, 44, 47	43, 44, 46, 47
46	84.4, qC			
47	74.4, CH	2.90, d, 2.0	45	44, 45

**Table 2:** ^1^H NMR (500 MHz, CD_3_OD) and ^13^C NMR (125 MHz, CD_3_OD) data of nepheliosyne **B** (**2**).

Position	δ_C_ (ppm)/Multiplicity	δ_H_ (ppm)/ *J* (Hz)/Multiplicity	^1^H-^1^H COSY/TOCSY	^1^H-^13^C HMBC
1	157.7, qC			
2	80.1, qC			
3	85.3, qC			
4	67.1, CH	4.26, d, 5.02	5, 6, 7	1, 5
5	74.9, CH	3.58, m	4, 6, 7	4
6a	33.5, CH_2_	1.69–1.64, m	4, 5, 7, 8	4, 5
6b	33.5, CH_2_	1.52–1.44, m	4, 5, 7, 8	4, 5
7	30.8–30.4, CH_2_	1.29, m	4, 5, 6, 8	8
8–18	30.8–30.4, CH_2_	1.29, m	5a, 5b, 7, 17, 19	
19	24.1, CH_2_	1.59–1.55, m	18, 20, 24, 25	20, 24, 25
20	38.6, CH_2_	2.61–2.49, m	18, 19, 25, 26	18, 19, 21
21	215.0, qC			
22	78.0, CH	4.05, dd, 8.1, 4.2	19, 23, 24	21, 23, 24
23a	34.6, CH_2_	1.57–1.50, m	22, 24, 25, 26	20
23b	34.6, CH_2_	1.73–1.67, m	22, 24, 25, 26	20
24	25.9, CH_2_	1.42–1.34, m	20, 22, 23, 25, 26	19, 23, 25, 26, 27
25	28.0, CH_2_	2.14–2.05, m	20, 23, 24, 26, 27	18, 19, 24, 26, 27
26	130.7, CH	5.38, m	20, 23, 24, 25, 28	25
27	130.9, CH	5.38, m	28, 29, 30, 31	28
28	27.8, CH_2_	2.14–2.05, m	26, 27, 29, 30, 31	26, 27, 29, 30
29	26.2, CH_2_	1.58–1.49, m	26, 27, 28, 30, 31	28, 30, 31
30	38.6, CH_2_	1.70–1.62, m	26, 27, 28, 29, 31	28, 29, 31
31	62.6, CH	4.33, dd, 6.7, 1.4	26, 27, 28, 29, 30, 34	30, 32, 33
32	83.1, qC			
33	82.9, qC			
34	52.4, CH	5.17, t, 1.6	29, 30, 31, 37, 38	32, 33, 35, 36
35	85.6, qC			
36	85.9, qC			
37	62.6, CH	4.36, dd, 6.6, 1.6	34, 38, 39, 40	35, 38
38	36.9, CH_2_	2.45, t, 6.1	31, 37, 39, 40	36, 37, 39, 40
39	127.6, CH	5.64–5.54, m	37, 38, 40, 41, 42	38, 42, 44
40	129.2, CH	5.64–5.54, m	37, 38, 39, 41, 42	41, 42, 44
41	36.4, CH_2_	2.38–2.25, m	39, 40, 42	39, 40, 42, 43
42	72.2, CH	4.15, dd, 6.3	39, 40, 41, 44	39, 40, 41, 43, 44
43	135.7, CH	5.91, ddd, 15.4, 5.9, 1.3	41, 42, 44, 45	42, 44, 45
44	130.7, CH	5.78, ddd, 15.4, 5.7, 1.2	41, 42, 43, 45	42, 43, 45, 46
45	62.6, CH	4.83, d, 5.5	37, 42, 43, 44, 47	43, 44, 46, 47
46	84.4, qC			
47	75.1, CH	2.90, d, 2.0	45	44, 45

Structural elucidations of the partial structures **b**–**e** hereafter are based on those of compound **1** as ^1^H and ^13^C NMR chemical shifts of these fragments are almost identical in both compounds.

Fragment **b** contained a ketone group flanked by one adjacent methylene at δ_H_ 2.61–2.49 (H_2_-20, δ_C_ 38.6) and an oxymethine at δ_H_ 4.05 (H_2_-22, δ_C_ 78.0), which are correlated to methylenes at δ_H_ 1.59–1.55 (H_2_-19, δ_C_ 24.1) and at δ_H_ 1.74–1.49 (H_2_-23, δ_C_ 34.6), respectively. 

Fragment **c** was constituted by the olefinic protons at δ_H_ 5.38 (H-26, δ_C_ 130.7 and H-27, δ_C_ 130.9), which are correlated to methylenes at δ_H_ 2.14–2.05 (H_2_-25, δ_C_ 28.0 and H_2_-28, δ_C_ 27.8). HMBC correlations were observed between these latter and H_2_-24 (δ_H_ 1.40–1.35 δ_C_ 26.2) and H_2_-29 (δ_H_ 1.56–1.49 δ_C_ 26.4), respectively.

Fragment **d** consisted of the oxymethines at δ_H_ 4.36 (H-37, δ_C_ 62.6) and δ_H_ 4.33 (H-31, δ_C_ 62.6), which correlated to the methylenes at δ_H_ 2.45 (H-38, δ_C_ 36.8) and at δ_H_ 1.70–1.64 (H_2_-30, δ_C_ 38.3), respectively. These protons showed a long-range coupling to the oxymethine located between two triple bonds (H-34, δ_H_ 5.17, δ_C_ 52.3). HMBC correlations were observed between C-32 (δ_C_ 83.1) and both H-31 (δ_H_ 4.33) and H-34 (δ_H_ 5.17), and between C-36 (δ_C_ 85.9) and methines at δ_H_ 4.36 (H-37) and H-34 (δ_H_ 5.17), whereas the signal at δ_C_ 82.9 (2C, C-33 and C-35) showed cross-peaks only with H-34 (δ_H_ 5.17) thus allowing the carbon assignment of the two triple bonds in the bis propargylic alcohol.

Fragment **e** was established to be formed sequentially by the acetylenic proton at δ_H_ 2.90 (H-47, δ_C_ 74.4), which constitutes the second terminus of the chain, the oxymethine at δ_H_ 4.83 (H-45, δ_C_ 62.5), the olefinic signals at δ_H_ 5.78 (H-44, δ_C_ 130.7) and δ_H_ 5.91 (H-43, δ_C_ 135.7), the oxymethine at δ_H_ 4.15 (H-42, δ_C_ 72.2), and the methylene at 2.38–2.25 (H_2_-41, δ_C_ 36.3). H_2_-41 is correlated to the olefinic protons at δ_H_ 5.64–5.54 (H-39, δ_C_ 129.2 and H-40, δ_C_ 127.5). In a similar way, HMBC correlations were observed between H-42 (δ_H_ 4.15) and C-41 (δ_C_ 36.3), C-43 (δ_C_ 135.7), and C-44 (δ_C_ 130.7).

The connectivities between these partial structures **a**–**e** for **1 **and **a**′–**e** for **2**, as well as the number of the linking methylene groups, were established on the basis of the ^1^H-^13^C HMBC, ^1^H-^1^H COSY/TOCSY correlations, and MS data. The correlation observed between H_2_-23 and H_2_-25 offered the connection between fragments **b** and **c**. In a similar way, the correlation between H_2_-28 and H_2_-30 provided the connection between the partial structures **c** and **d**. Finally, the correlation between H-37 and H-39 offered the connection between the partial structures **d** and **e**. The combinations **a** + **b** + **c** + **d** + **e** and **a′** + **b** + **c** + **d** + **e** represented 670 m.u. whereas the molecular structure weight was 810 m.u. The difference corresponding to 10 methylene groups determined the length of the complementary alkyl chain between **a** (or **a′**) and **b**. All the spectral data of **1** (1D and 2D NMR, MS, and optical properties) led to its identification as nepheliosyne **A**, in accordance with previous published data [3]. Thus, nepheliosyne **B** (**2**) is a new metabolite defined as the 5-hydroxy-6-dehydroxy derivative of nepheliosyne **A** (**1**).

The geometry of the double bond Δ^43,44^ was easily assigned as *E* by analysis of the coupling constant of the olefinic protons (*J*_43,44_ = 15.5 Hz). The geometries of the double bond Δ^26,27^ and Δ^39,40^ were assigned as *Z* and *E*, respectively, based on the ^13^C chemical shifts of the allylic methylenes δ_C_ 28.0 (C-25) and 27.8 (C-28) for Δ^26,27^ and δ_C_ 36.8 (C-38) and 36.3 (C-41) for Δ^39,40^.

The relative and absolute configurations of the stereogenic centers of nepheliosyne **A** (**1**) and **B** (**2**) remained unassigned. They were observed also to degrade rapidly under different reaction conditions [14,15,16]. Like fulvynes [12], any attempts to obtain suitable derivatives for a stereochemical analysis were unsuccessful. 

To date, several polyhydroxylated acetylenic metabolites of marine sponges with a diacetylenic carbinol and a α-yne carboxylic group have been reported: Nepheliosyne **A** from *Xestospongia* sp. [3], petrosolic acid from *Petrosia* sp. [6], osirisynes [10] and haliclonyne [11] from *Haliclona* sp., and fulvynes **A**–**I** from *Haliclona fulva* [12]. Nepheliosyne **B** (**2**) is, with nepheliosyne **A** and petrosolic acid, the third example of linear acetylene with diacetylenic carbinol, α-hydroxyketone, and α-yne carboxylic groups. To the best of our knowledge this is the first report on the isolation and structure identification of oxygenated polyacetylenic metabolites from *Niphates* sp. All these data suggest that from a chemotaxonomic point of view polyhydroxylated acetylenic metabolites could constitute potential markers of Haplosclerida species.

The cytotoxicity of the nepheliosynes **A** (**1**) and **B** (**2**) was evaluated against K562, U266, SKM1, and Kasumi cancer cell lines which are widely used for cytotoxicity assays, and the IC_50_ values in μM (XTT assay) are indicated in Table 3. Compounds **1** and **2** were equally efficient with IC_50_ values around 150–200 μM. Interestingly, their significant specificity against tumor cells were highlighted thanks to additional assays showing that, compared to the K562 cells, the peripheral blood mononuclear cells (PBMC) viability is not affected by compounds **1** and **2** (see Supporting Information).

**Table 3:** IC_50_ values for compounds **1** and **2** for loss of cell metabolism (XTT assay) and cell number. Cells (10 × 10^4^/mL) were incubated for 48 h at 37 °C with either increasing concentrations of compounds **1** and **2** in the 2.5–250 μM range. Cell metabolism was measured using the XTT kit assay as indicated in the Experimental section. IC_50_ values are representative of three experiments made in quadruplicates.

Compounds	SKM1 (IC_50_)	U266 (IC_50_)	Kasumi (IC_50_)	K562 (IC_50_)
1	250	170	200	200
2	>250	200	150	150

## 3. Experimental Section

### 3.1. General

All organic solvents used for material extraction were of analytical grade and purchased from Merck (Darmstadt, Germany). Acetonitrile used for HPLC was of HPLC-grade and purchased from Fisher Scientific, USA. Formic acid of HPLC grade was purchased from Acros, USA. 2,5-Dihydroxybenzoic acid (DHB), used as the matrix for MALDI-TOF experiments, was of the highest grade available and used without further purification was purchased from Sigma Aldrich Co, France. The Chromabond C18 preparative column used for flash chromatography was obtained from Merck, USA. UV measurements were performed on a Varian Cary 300 Scan UV-visible spectrometer. IR spectra were obtained with a Perkin-Elmer Spectrum 100 series FT-IR spectrometer equipped with an universal attenuated total reflectance sampling accessory (ATR). Optical rotations were measured on a Jasco P-1010 polarimeter. Flash chromatography was performed on an Armen Instrument Spot Liquid Chromatography system, the detection wavelength was set at 254 nm. HPLC purifications were carried out on a Waters 600 system equipped with a Waters 717 plus autosampler, a Waters 996 photodiode array detector, and a Sedex 55 evaporative light-scattering detector (SEDERE, Alfortville, France). Detection wavelengths were set at 214, 254 and 280 nm. ^1^H and ^13^C NMR spectra were recorded with 500 MHz Bruker Avance NMR spectrometers. Chemical shifts (δ) are recorded in ppm with CD_3_OD (δ_H_ 3.31 ppm and δ_C_ 49.0 ppm) as internal standard with multiplicity (s singlet, d doublet, t triplet, m multiplet). High resolution mass spectra (HRMS) were conducted on a Voyager DE-STR MALDI-TOF mass spectrometer (ABSciex, Les Ulis, France), equipped with a 337 nm pulsed nitrogen laser (20 Hz) and a Acquiris^®^ 2 GHz digitizer board, was used for all experiments. Mass spectra were obtained in reflectron positive ion mode with the following settings: Accelerating voltage 20 kV, grid voltage 62% of accelerating voltage, extraction delay time of 100 ns. The laser intensity was set just above the ion generation threshold to obtain peaks with the highest possible signal-to-noise (S/N) ratio without significant peak broadening. All data were processed using the Data Explorer software package (ABSciex).

### 3.2. Sponge Material

**Figure 3:**
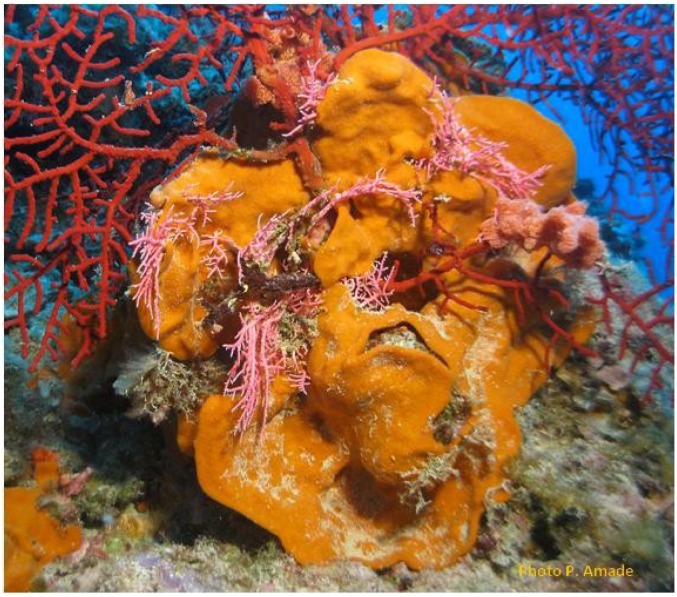
*Niphates* sp. (photo: Philippe Amade).

We collected the sponge *Niphates* sp. (Schmidt, 1862) (Demospongiae, Haplosclerida, Niphatidae) in November 2008 using scuba at a depth of 22 m in the south-west lagoon of New Caledonia (Philippe Amade, Figure 3). The sponge was identified by J. Vacelet and a voucher specimen (MHNM 1646) was deposited at the Natural History Museum of Marseille (France) [17].

### 3.3. Extraction and Isolation

A portion of *Niphates* sp. was freeze-dried and ground to obtain a dry powder (28 g), which was exhaustively extracted with a mixture of MeOH/CH_2_Cl_2_ (1:1, v/v) to yield 463 mg of the crude extract after concentration under reduced pressure. The crude extract was fractionated by RP-C18 flash chromatography (elution with a decreasing polarity gradient of H_2_O/MeOH from 1:0 to 0:1, then MeOH/CH_2_Cl_2_ from 1:0 to 0:1) (flow: 10 mL・min^−1^). The MeOH fraction (100 mg) was then subjected to semi-preparative HPLC-DAD (Phenomenex Luna C18, 250 × 10 mm id, 5 μm) with a gradient of H_2_O/MeCN/Formic acid 50:50:0.1 to 0:100:0.1 (flow: 3.0 mL・min^−1^, injection volume: 100 μL) to afford the pure compounds **1** and **2**. Both were identified by a combination of spectroscopic methods (1D and 2D NMR, MS) and comparison with the literature data [3,4,5,6,7,8,9,10,11,12].

### 3.4. Characterization Data

Nepheliosyne **A**: amorphous solid; [α]_D_ = +7.0 (*c* 0.01, MeOH); IR (solid) 3450, 3300, 2250, 2100, 1705 cm^−1^; UV λ_max_ (MeOH) 205 nm (ε 2500); HR-MALDITOF-MS *m*/*z* 833.4803 [M + Na]^+^ (Calcd. for C_47_H_70_O_11_Na 833.4726, Δ = −1.05 ppm); For ^1^H NMR (500 MHz) and ^13^C NMR (125 MHz) data see Table 1.

Nepheliosyne **B**: amorphous solid; [α]_D_ = +9.9 (*c* 0.01, MeOH); IR (solid) 3450, 3300, 2250, 2100, 1705 cm^−1^; UV λ_max_ (MeOH) 205 nm (ε 2500); HR-MALDITOF-MS *m*/*z* 833.4803 [M + Na]^+^ (Calcd. for C_47_H_70_O_11_Na 833.4726, Δ = −1.05 ppm); For ^1^H NMR (500 MHz) and ^13^C NMR (125 MHz) data see Table 2.

### 3.5. Biological Activity

Cell Lines. The human cancer cell lines K562 (chronic myelogenous leukemia), U266 (myeloma), SKM1 (myelodysplastic symdrom), and Kasumi (acute myeloid leukemia) were provided by American Type Culture Collection (ATCC) and were grown at 37 °C under 10% CO_2_ in RPMI 1640 medium (Gibco BRL, Paisley, UK) supplemented with 10% Fetal Calf Serum (FCS) (Gibco BRL, Paisley, UK) completed with 50 units/mL penicillin, 50 mg/mL streptomycin and 1 mM sodium pyruvate. 

Measurement of Cell Metabolism (XTT). Cells (10 × 10^4^/mL) were incubated with **1** or **2** for the times indicated. 50 μL of XTT kit (sodium 39-[1-(phenylaminocarbonyl)-3,4-tetrazolium]-bis(4-methoxy-6-nitro)benzene sulfonic acid hydrate) was added to each well, which contain 100 μL of medium. Absorbance of the formazan dye produced by metabolically active cells was measured at 490 nm as described earlier [18]. Each assay was performed in quadruplicate. 

## 4. Conclusions

Nepheliosyne **B** (**2**), a new C47 highly oxygenated polyacetylene, along with nepheliosyne **A** (**1**), have been isolated from the New Caledonian marine sponge *Niphates* sp. Their structures have been determined on the basis of extensive spectroscopic analyses which led us to complete the structure determination of the previously reported nepheliosyne **A** (**1**). With a significant specificity, these metabolites exhibited a moderate cytotoxicity against K562, U266, SKM1, and Kasumi cancer cell lines.

## Supplementary Files

Supplementary File 1 Supplemental Information (PDF, 1093 KB)
