# Supplementary material for: Nepheliosyne B, a New Polyacetylenic Acid from the New Caledonian Marine Sponge Niphates sp
Source: Mar Drugs. 2013 Jun 27;11(7):2282–92. doi: 10.3390/md11072282 (PMC3736423; doi:10.3390/md11072282)

Supplemental Information

Figure S1. ^{1}H NMR spectrum of nepheliosyne **A** (**1**) in CD_3OD at 500 MHz............2
Figure S2. ^{13}C NMR spectrum of nepheliosyne **A** (**1**) in CD_3OD at 125 MHz............3
Figure S3. ^{1}H-^{1}H COSY spectrum of nepheliosyne **A** (**1**) in CD_3OD............4
Figure S4. ^{1}H-^{13}C HSQC spectrum of nepheliosyne **A** (**1**) in CD_3OD............5
Figure S5. ^{1}H-^{13}C HMBC spectrum of nepheliosyne **A** (**1**) in CD_3OD............6
Figure S6. ^{1}H-^{1}H TOCSY spectrum of nepheliosyne **A** (**1**) in CD_3OD............7
Figure S7. ^{1}H NMR spectrum of nepheliosyne **B** (**2**) in CD_3OD at 500 MHz............8
Figure S8. ^{13}C NMR spectrum of nepheliosyne **B** (**2**) in CD_3OD at 125 MHz............9
Figure S9. ^{1}H-^{1}H COSY spectrum of nepheliosyne **B** (**2**) in CD_3OD............10
Figure S10. ^{1}H-^{13}C HSQC spectrum of nepheliosyne **B** (**2**) in CD_3OD............11
Figure S11. ^{1}H-^{13}C HMBC spectrum of nepheliosyne **B** (**2**) in CD_3OD............12
Figure S12. ^{1}H-^{1}H TOCSY spectrum of nepheliosyne **B** (**2**) in CD_3OD............13
Figure S13. Dose-response effect of nepheliosynes **A** (**1**) and **B** (**2**) on PBMC cells............14

Figure S1. ^{1}H NMR spectrum of nepheliosyne **A** (**1**) in CD_3OD at 500 MHz.

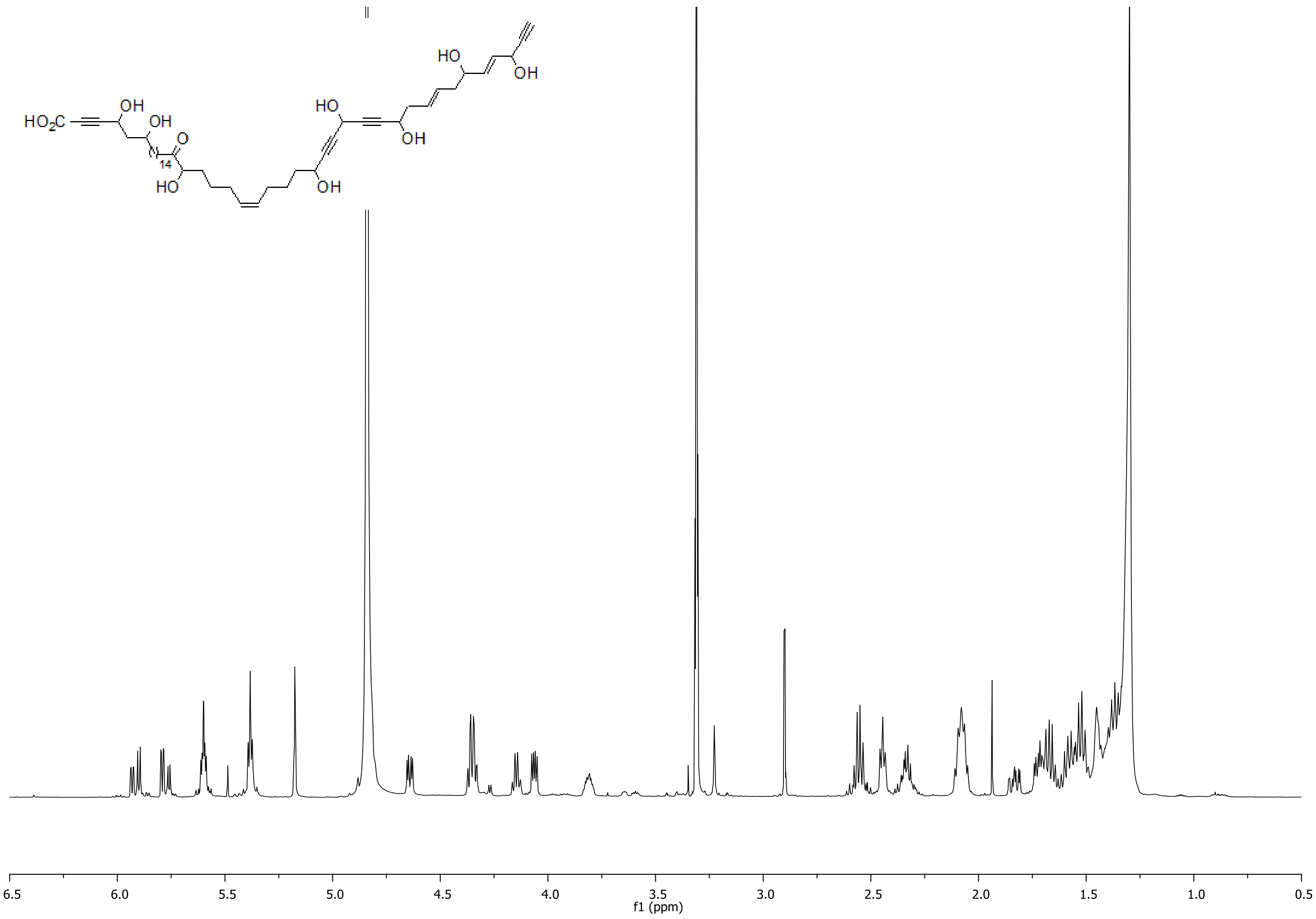

Figure S2. ^{13}C NMR spectrum of nepheliosyne **A** (**1**) in CD_3OD at 125 MHz.

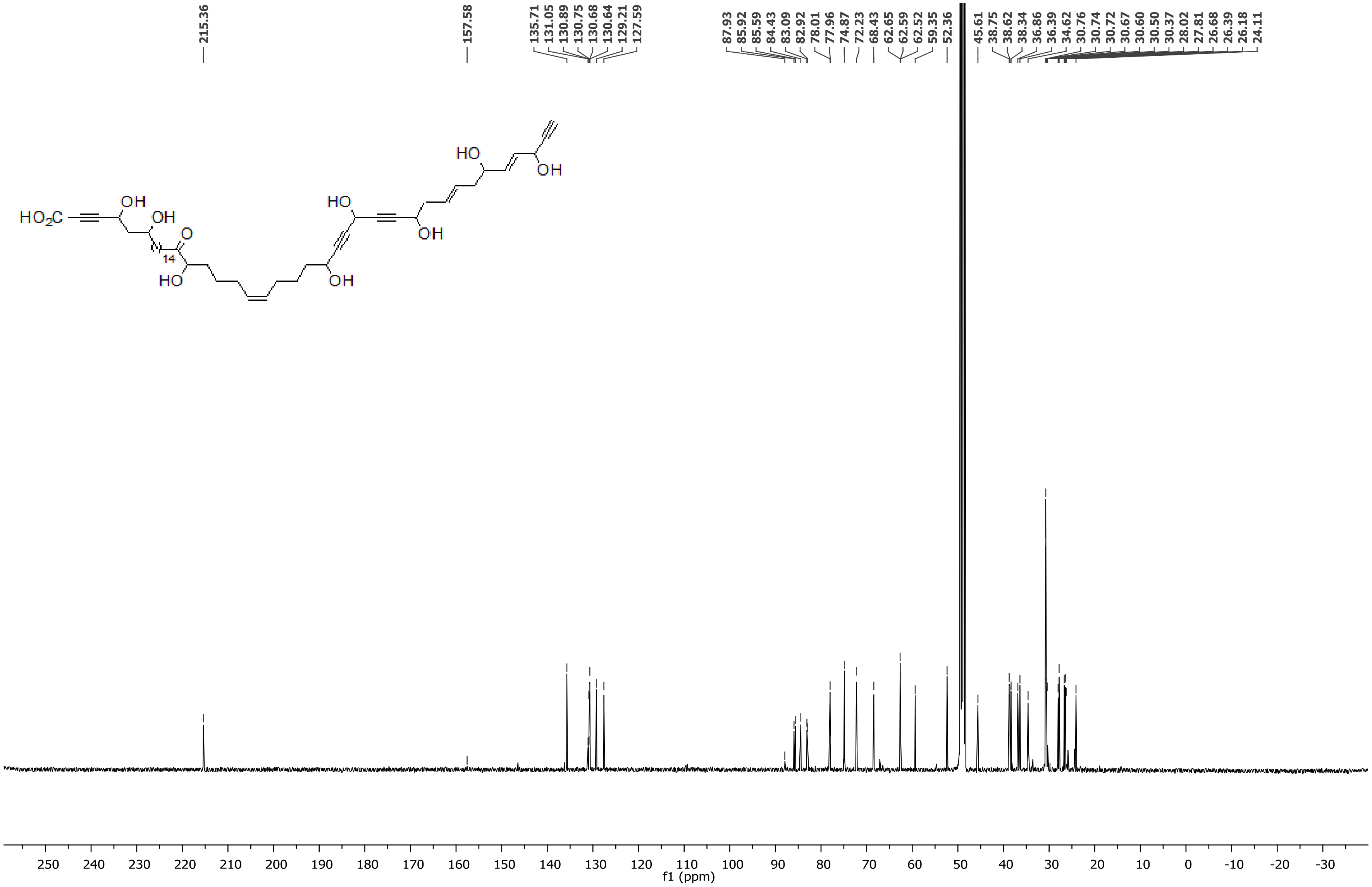

Figure S3. ^{1}H-^{1}H COSY spectrum of nepheliosyne **A** (**1**) in CD_3OD.

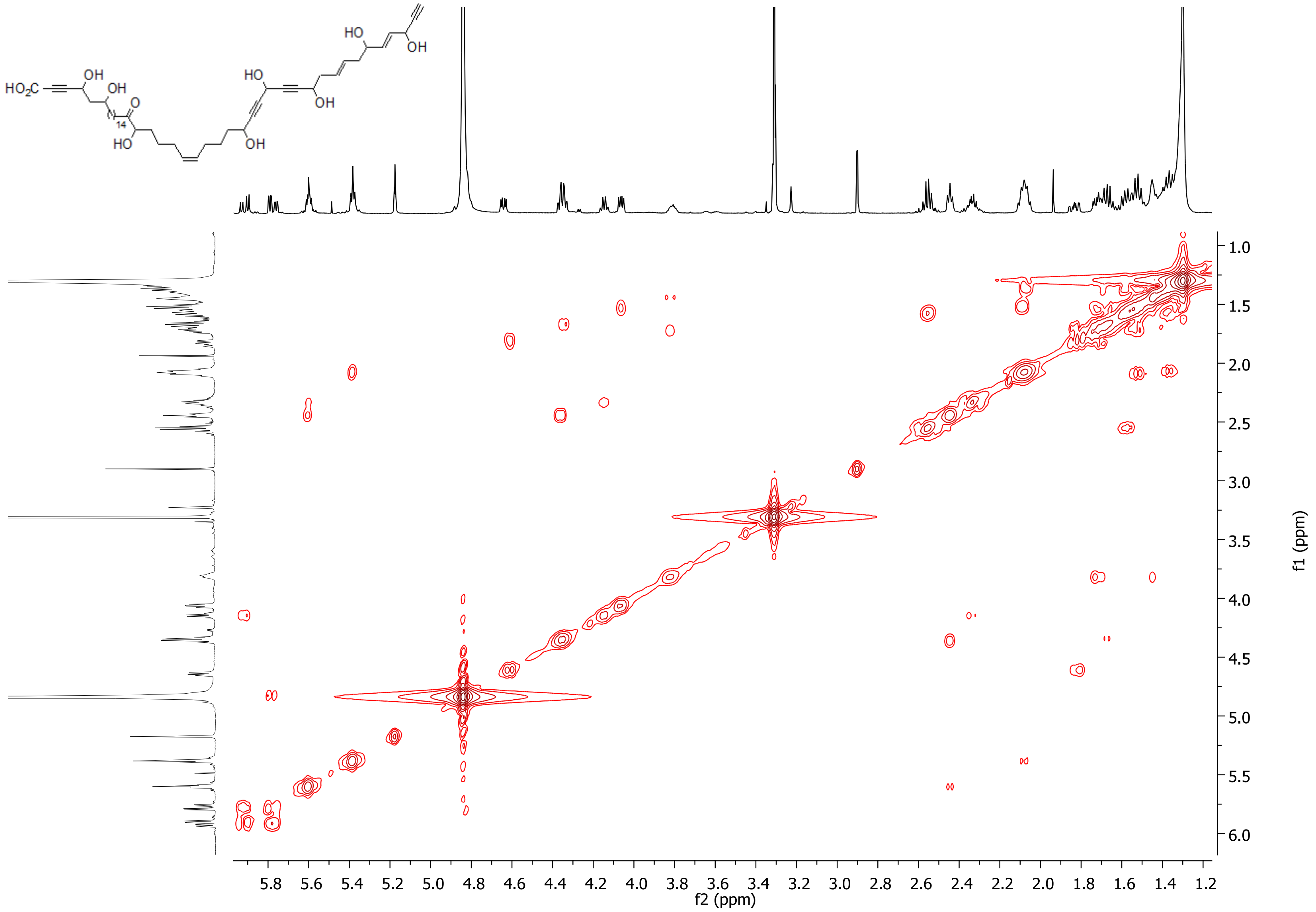

Figure S4. ^{1}H-^{13}C HSQC spectrum of nepheliosyne **A** (**1**) in CD_3OD.

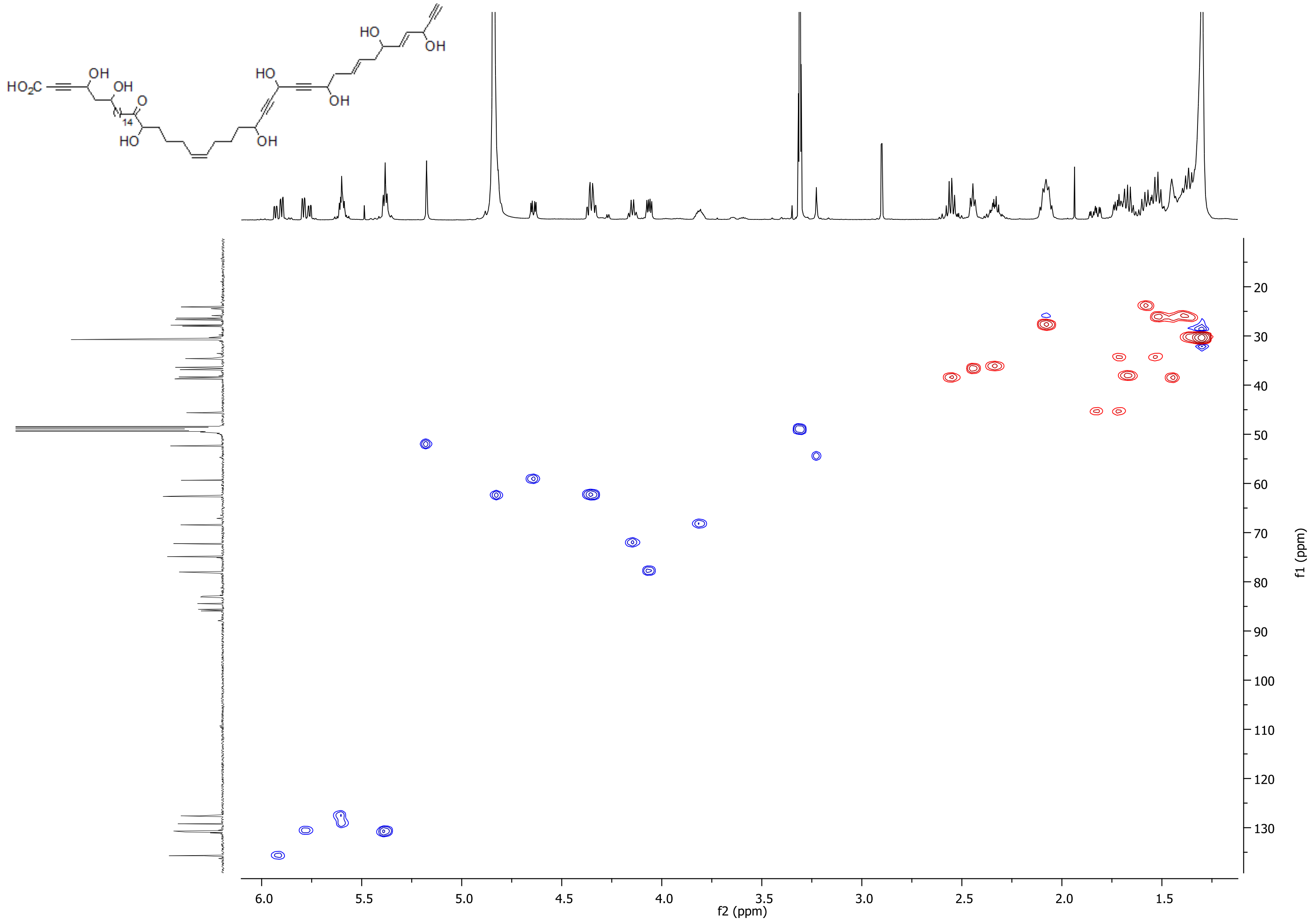

Figure S5. ^{1}H-^{13}C HMBC spectrum of nepheliosyne **A** (**1**) in CD_3OD.

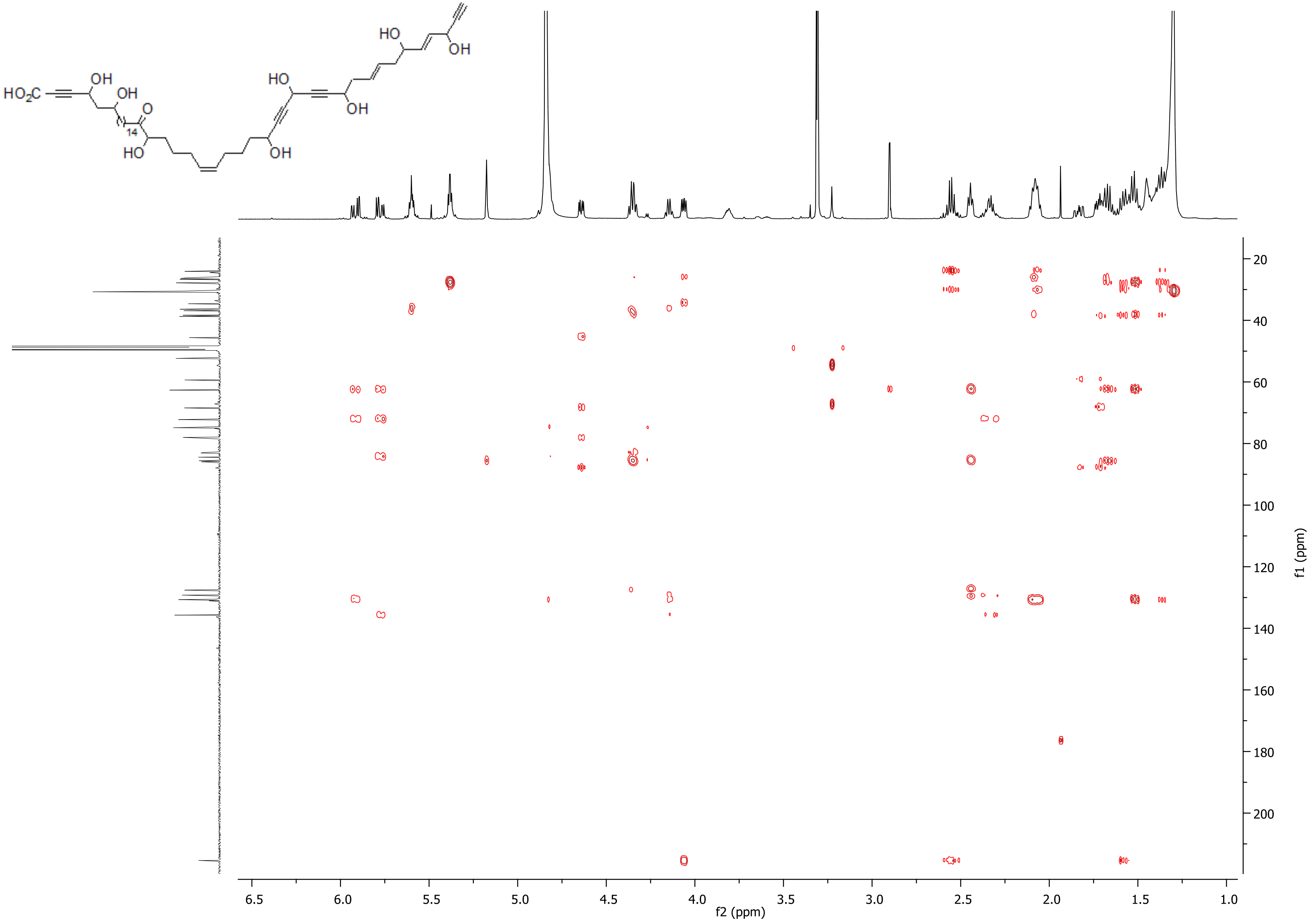

Figure S6. ^{1}H-^{1}H TOCSY spectrum of nepheliosyne **A** (**1**) in CD_3OD.

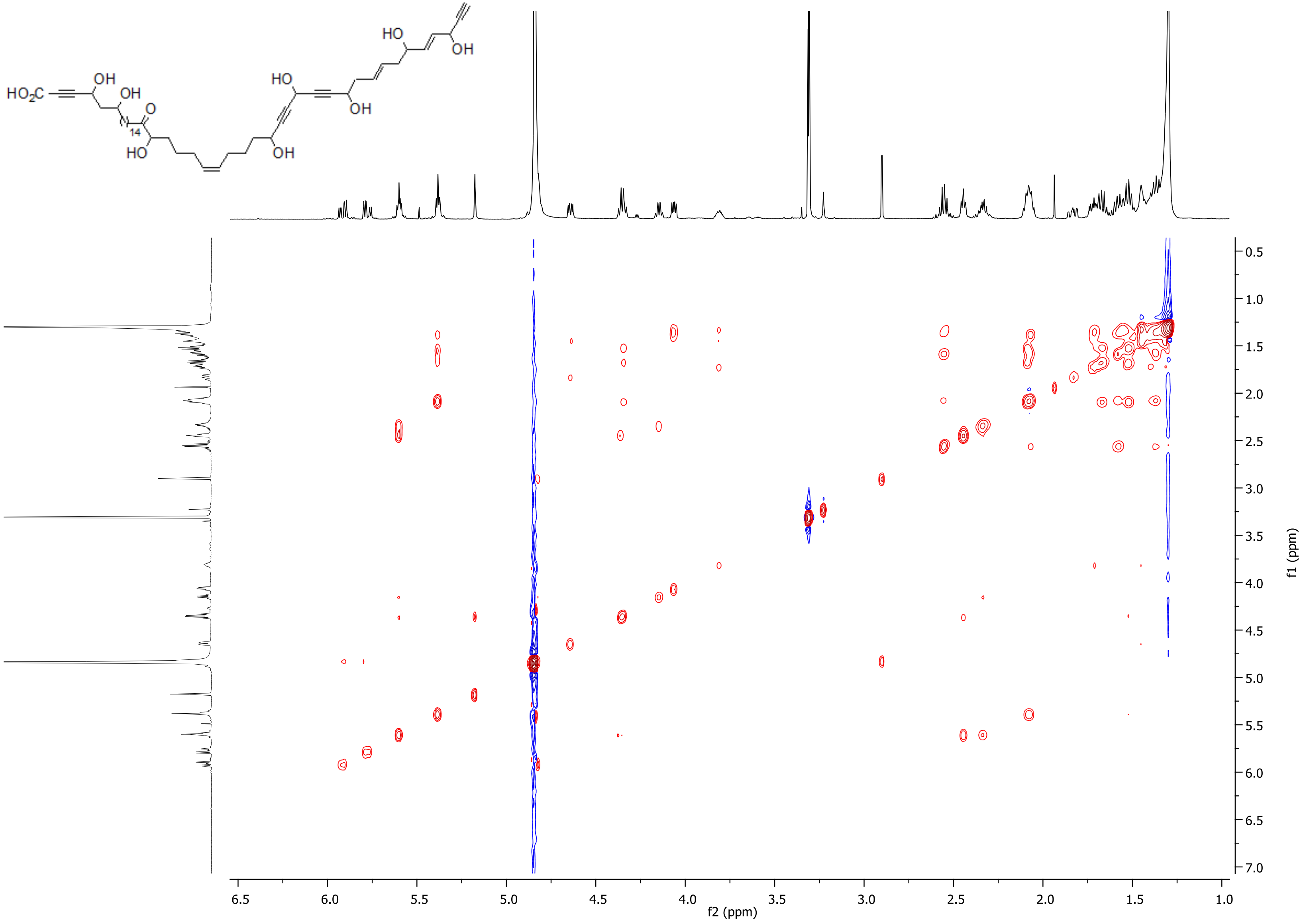

Figure S7. ^{1}H NMR spectrum of nepheliosyne **B** (**2**) in CD_3OD at 500 MHz.

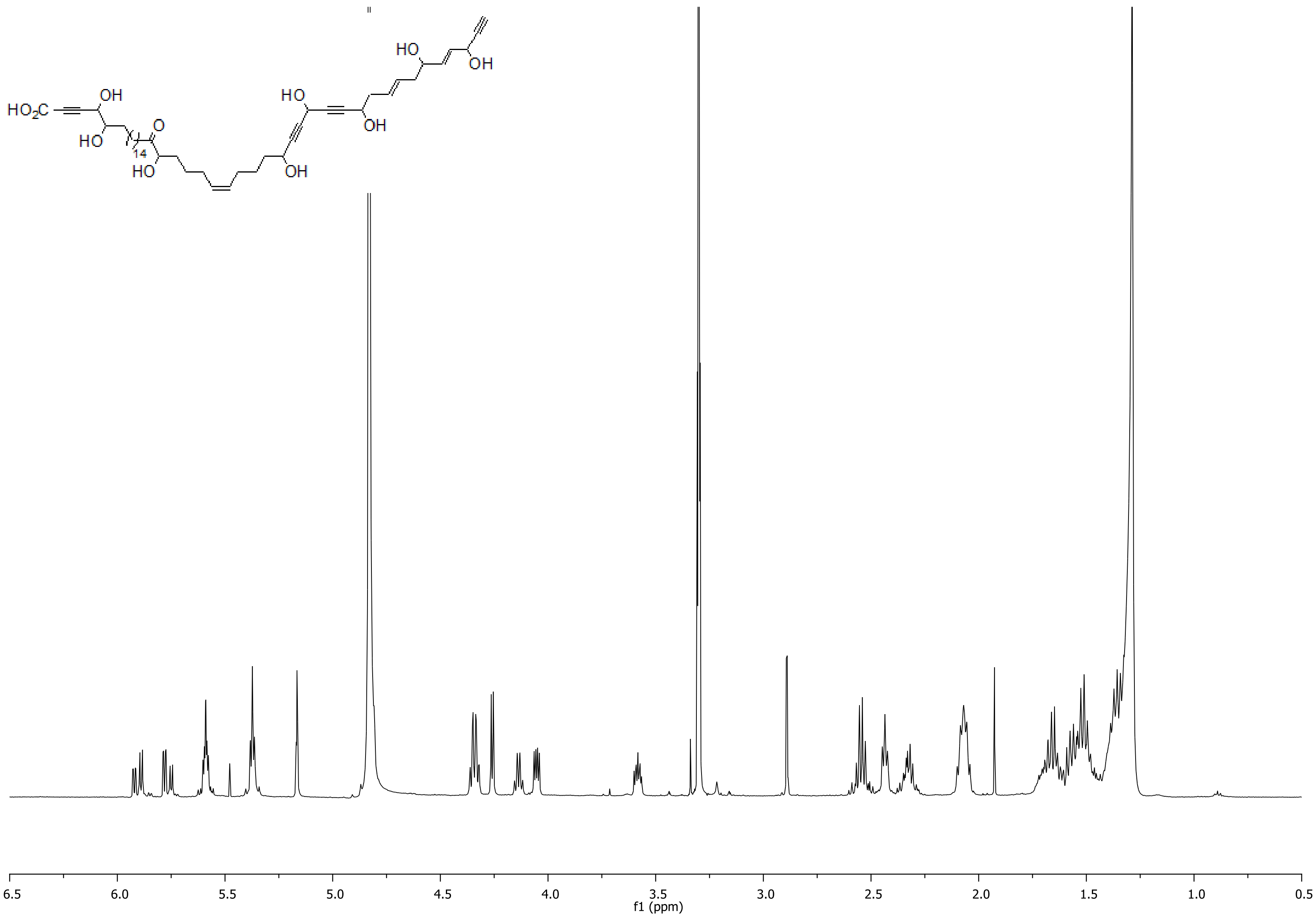

Figure S8. ^{13}C NMR spectrum of neph eliosyne **B** (**2**) in CD_3OD at 125 MHz.

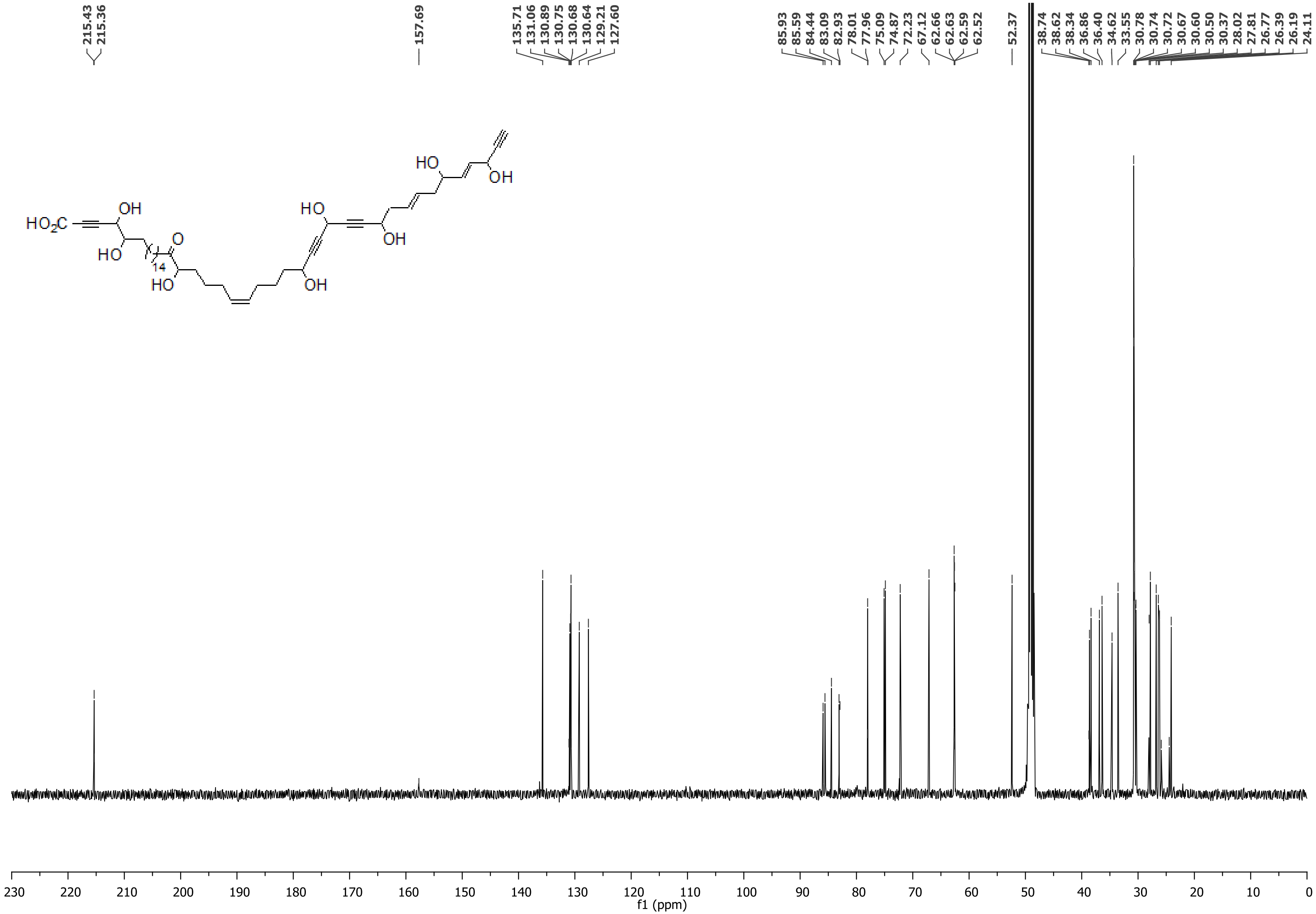

Figure S9. ^{1}H-^{1}H COSY spectrum of nepheliosyne **B** (**2**) in CD_3OD.

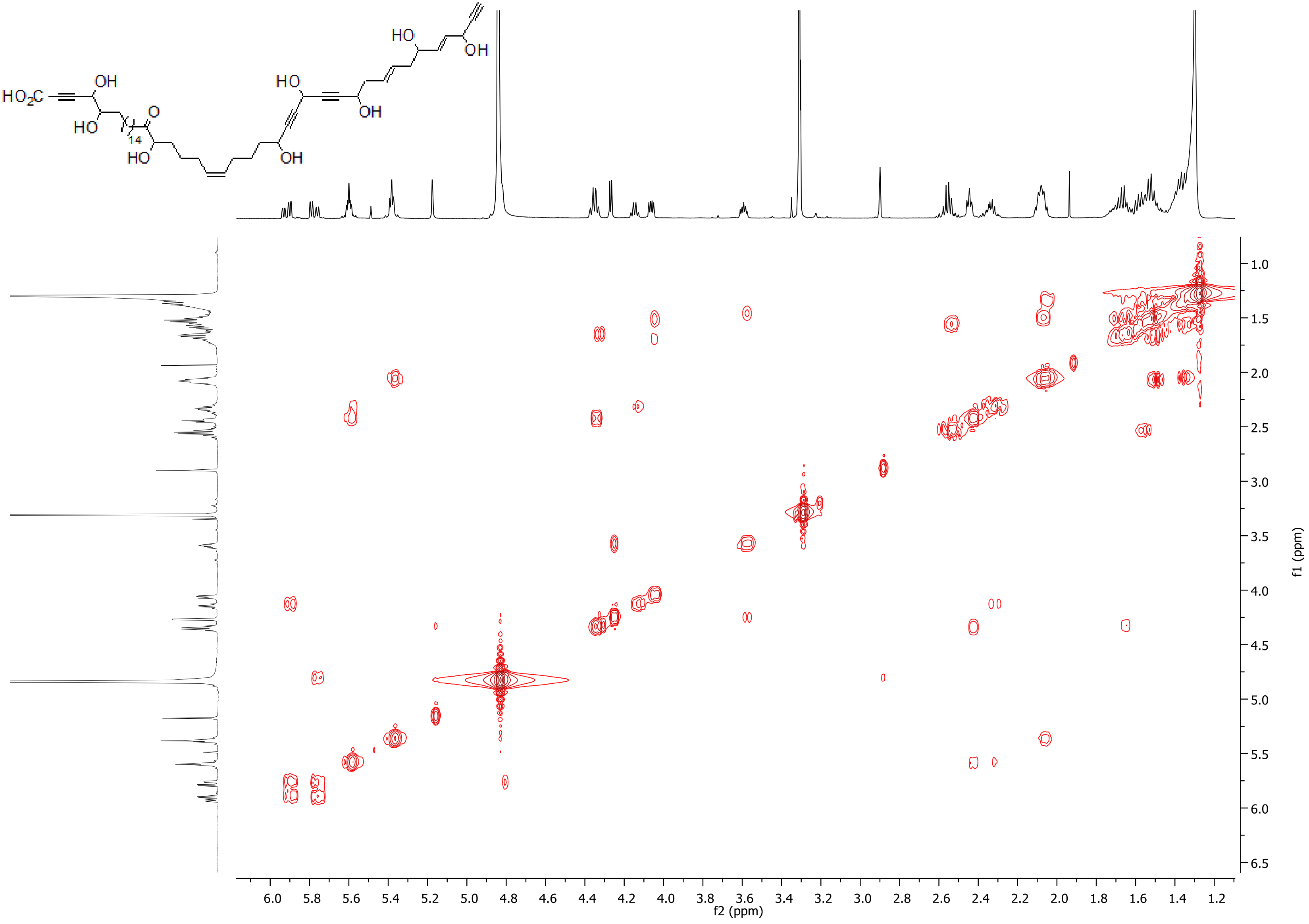

Figure S10. ^{1}H-^{13}C HSQC spectrum of nepheliosyne **B** (**2**) in CD_3OD.

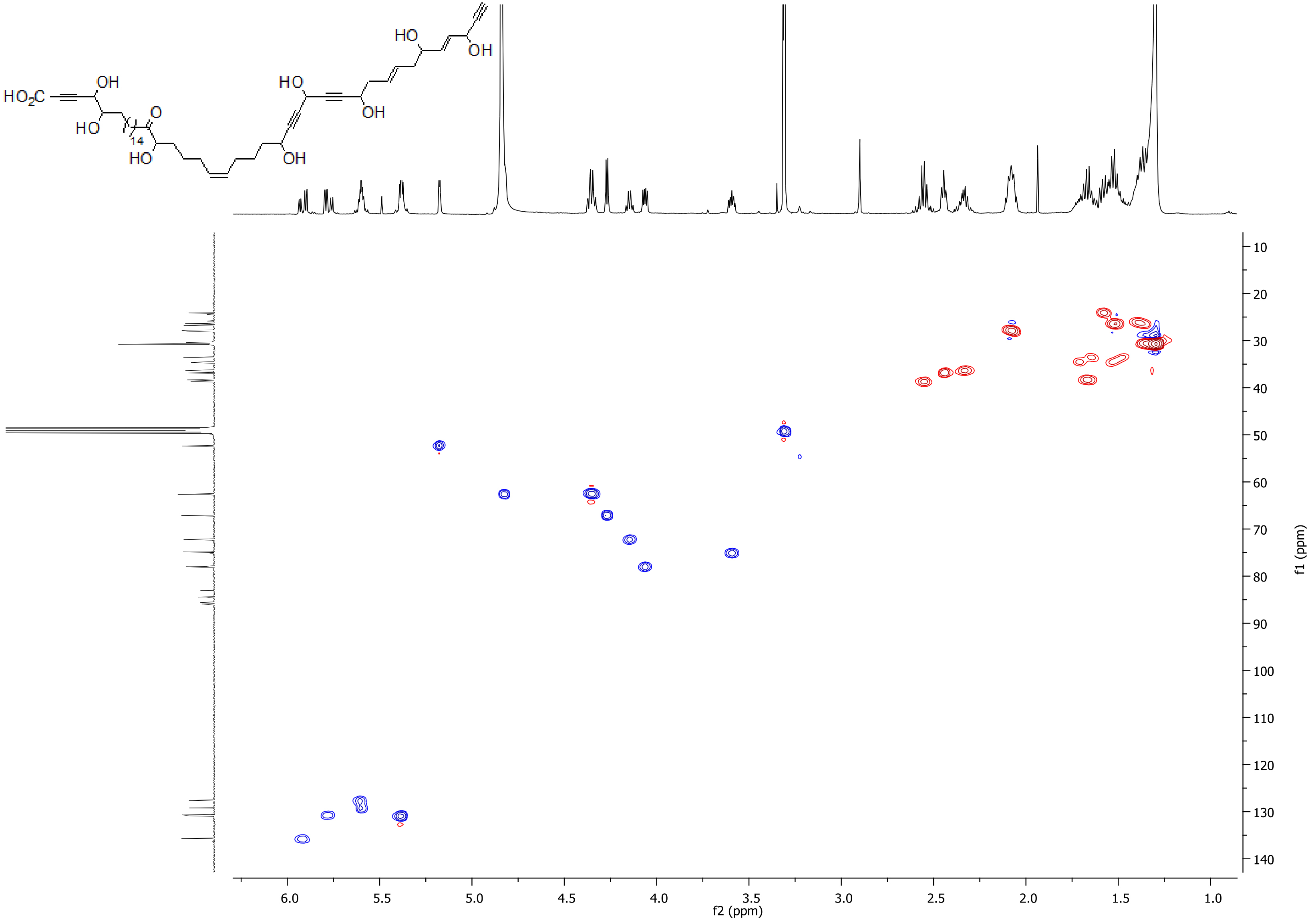

Figure S11. ^{1}H-^{13}C HMBC spectrum of nepheliosyne **B** (**2**) in CD_3OD.

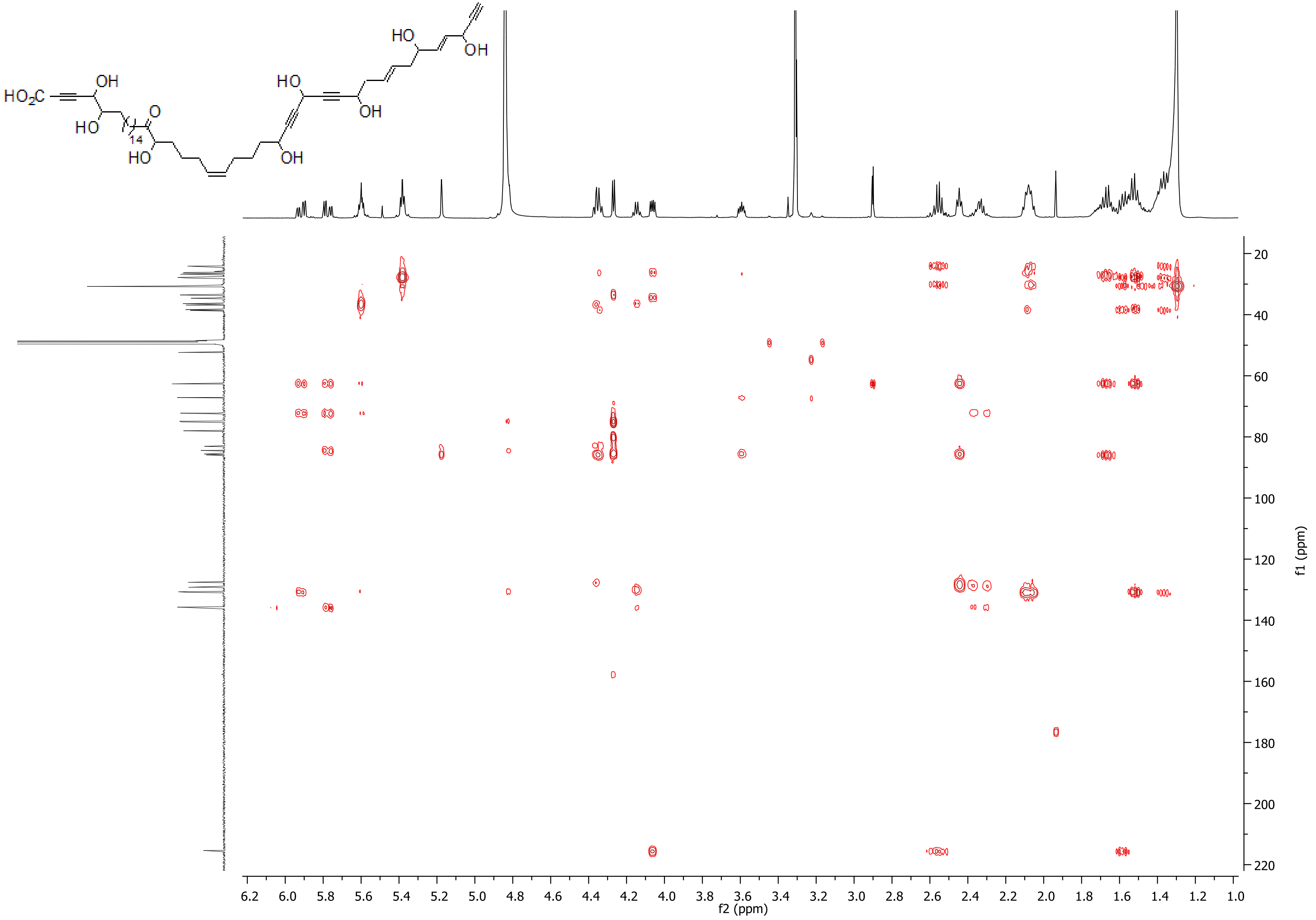

Figure S12. ^{1}H-^{1}H TOCSY spectrum of nepheliosyne **B** (**2**) in CD_3OD.

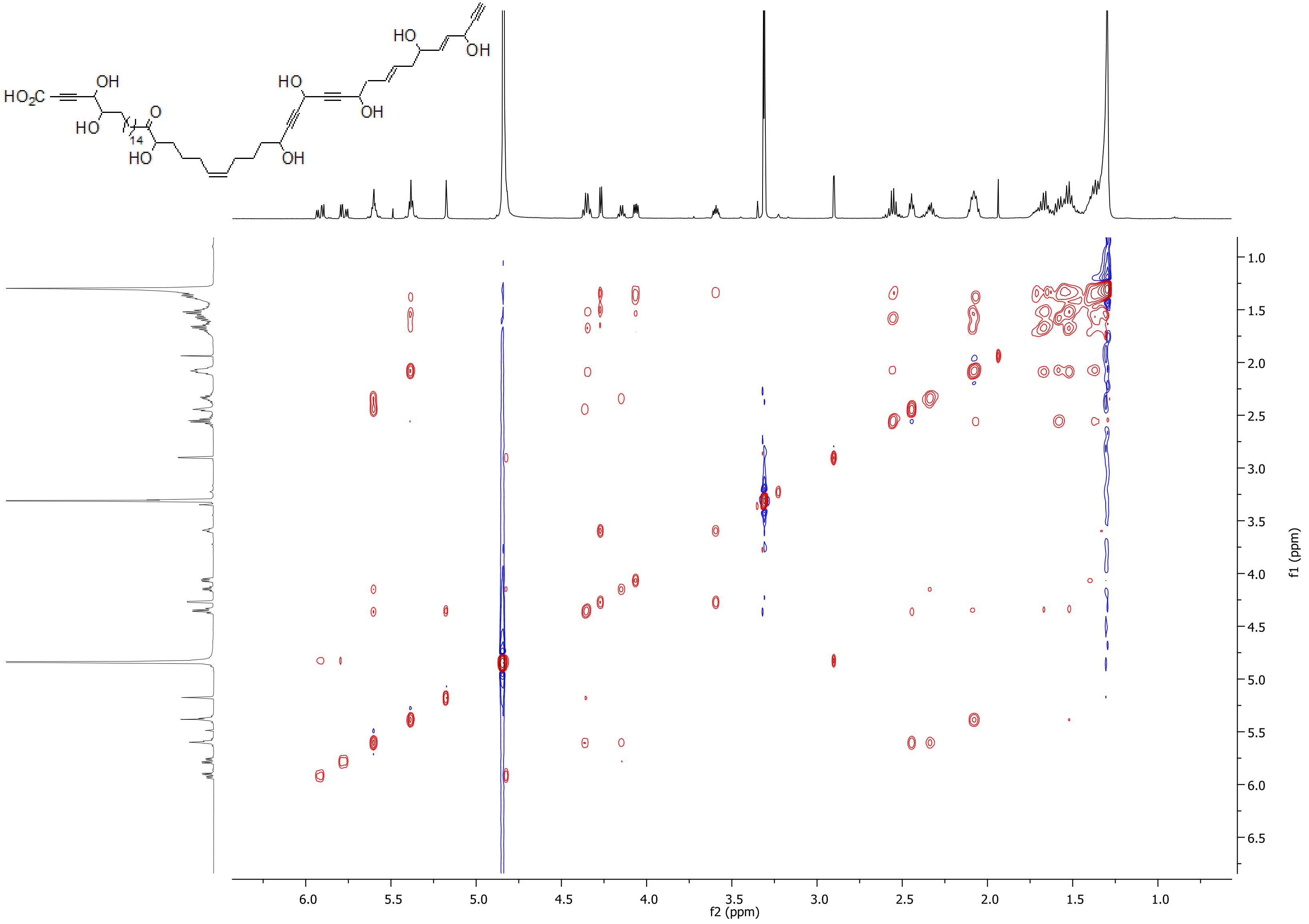

Figure S13. Dose-response effect of nepheliosynes **A** (**1**) and **B** (**2**) on PBMC cells.

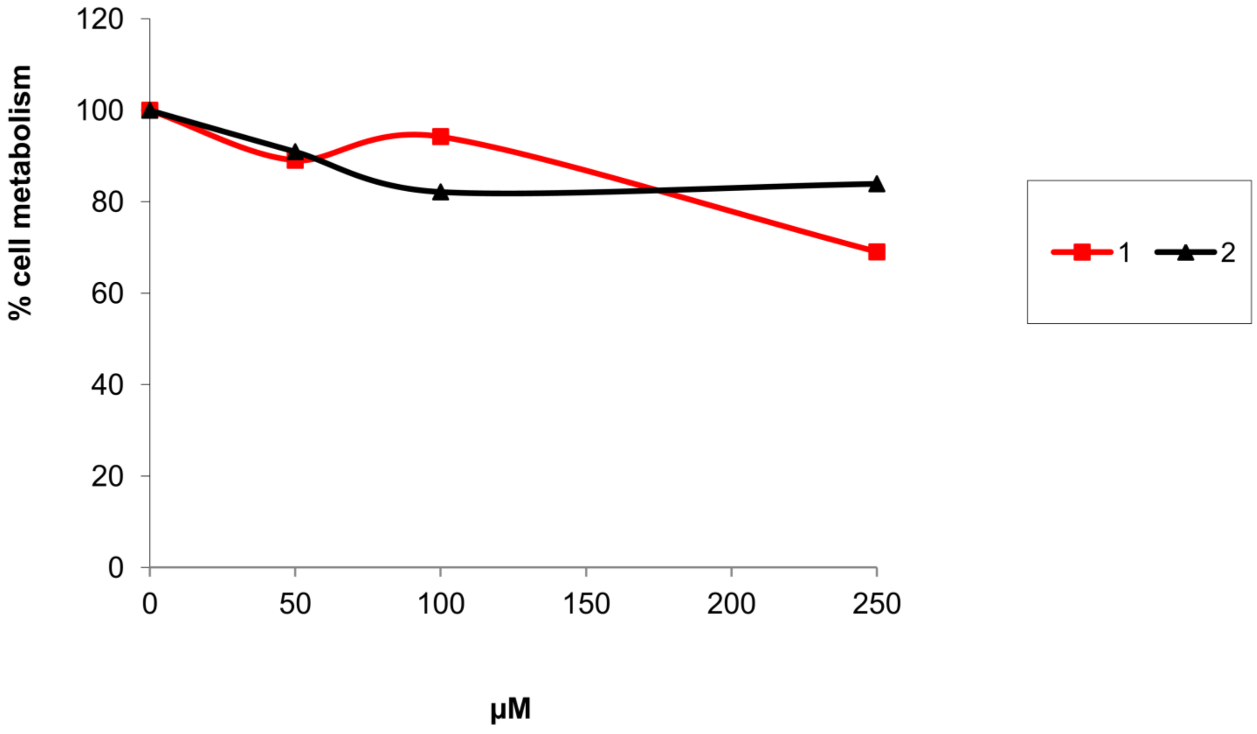

Supplement: Supplementary File 1 — Supplemental Information (PDF, 1093 KB) [file marinedrugs-11-02282-s001.pdf]
